# An immune-focused supplemental alignment pipeline captures information missed from dominant single-cell RNA-seq analyses, including allele-specific MHC-I regulation

**DOI:** 10.3389/fimmu.2025.1596760

**Published:** 2025-08-08

**Authors:** Sebastian Benjamin, GW McElfresh, Maanasa Kaza, Gregory J. Boggy, Benjamin Varco-Merth, Sohita Ojha, Shana Feltham, William Goodwin, Candice Nkoy, Derick Duell, Andrea Selseth, Tyler Bennett, Aaron Barber-Axthelm, Nicole N. Haese, Helen Wu, Courtney Waytashek, Carla Boyle, Jeremy V. Smedley, Caralyn S. Labriola, Michael K. Axthelm, R. Keith Reeves, Daniel N. Streblow, Jonah B. Sacha, Afam A. Okoye, Scott G. Hansen, Louis J. Picker, Benjamin N. Bimber

**Affiliations:** ^1^ Oregon National Primate Research Center, Oregon Health and Science University, Beaverton, OR, United States; ^2^ Vaccine and Gene Therapy Institute, Oregon Health and Science University, Beaverton, OR, United States; ^3^ Division of Innate and Comparative Immunology, Center for Human Systems Immunology, Duke University School of Medicine, Durham, NC, United States; ^4^ Department of Surgery, Duke University School of Medicine, Durham, NC, United States

**Keywords:** single-cell RNA-seq (scRNA-seq), T cells, bioinformatics, immunogenetics, major histocompatability complex (MHC)

## Abstract

**Introduction:**

RNA sequencing (RNA-seq) can measure whole transcriptome gene expression from tissues or even individual cells, providing a powerful tool to study the immune response. Analysis of RNA-seq data involves mapping relatively short sequence reads to a reference genome, and quantifying genes based on the position of alignments relative to annotated genes. While this is usually robust, genetic polymorphism or genome/annotation inaccuracies result in genes with systematically missing or inaccurate data. These issues are frequently hidden or ignored, yet are highly relevant to immunologic data, where balancing selection has generated many polygenic gene families not accurately represented in a ‘one-size-fits-all’ reference genome.

**Methods:**

Here we present nimble, a tool to supplement standard RNA-seq pipelines. Nimble uses a previously developed pseudoaligner to process either bulk- or single-cell RNA-seq data using custom gene spaces. Importantly, nimble can apply customizable scoring criteria to each gene set, tailored to the biology of those genes.

**Results:**

We demonstrate that nimble recovers data in diverse contexts, ranging from simple cases (e.g., incorrect gene annotation or viral RNA), to complex immune genotyping (e.g., major histocompatibility or killer-immunoglobulin-like receptors). We use this enhanced capability to identify killer-immunoglobulin-like receptor expression specific to tissue-resident memory T cells and demonstrate allele-specific regulation of MHC alleles after *Mycobacterium tuberculosis* stimulation.

**Discussion:**

Combining nimble data with standard pipelines enhances the fidelity and accuracy of experiments, maximizing the value of expensive datasets, and identifying cellular subsets not possible with standard tools alone.

## Introduction

RNA-sequencing (RNA-seq) and single-cell RNA-sequencing (scRNA-seq) technologies provide transcriptome-wide quantification in a sample of interest. In the case of scRNA-seq, transcriptomes are captured from individual cells, allowing for high-resolution observations of cellular function and differentiation. These high-dimensional data benefit the analysis of large populations of cells, such as those common in immunologic data. The rapid and accurate production of these data relies on complex software quantification toolchains. The process of transcript quantification is characterized by many technical decision points which, while generally obscured from downstream analysis, have a profound impact on the produced count data, depending on the quantification method of choice and its interaction with the reference genome.

The bioinformatic processing of RNA-seq and scRNA-seq data involves several steps. In most cases, short reads are aligned to a reference genome which is annotated for gene and features. In general, one genome is used to represent the diversity of the entire species. After alignment, an algorithm is run to assign reads to genes/features, producing gene counts. There are many established tools and pipelines for RNA-seq analysis. STAR is a commonly used alignment tool that can align reads by local positional alignment to a reference genome or transcriptome in a splice-aware manner (1). Kallisto performs transcript quantification by pseudoalignment of reads to a reference genome, without undergoing an expensive positional alignment process first (2). Feature calling is sometimes included with the aligner, and is sometimes performed using a separate tool, such as HTseq (3). Especially for scRNA-seq analyses, it is common for vendors to wrap all steps into one pipeline, such as the 10x Genomics CellRanger software. While these tools have differences in their implementation, they each function by aligning all data from a sample to a single reference genome, and they score genes/features using a ‘one-size-fits-all’ logic that treats all genes identically. This approach can work quite well and is probably the desirable approach for most genes.

**Figure 1:**
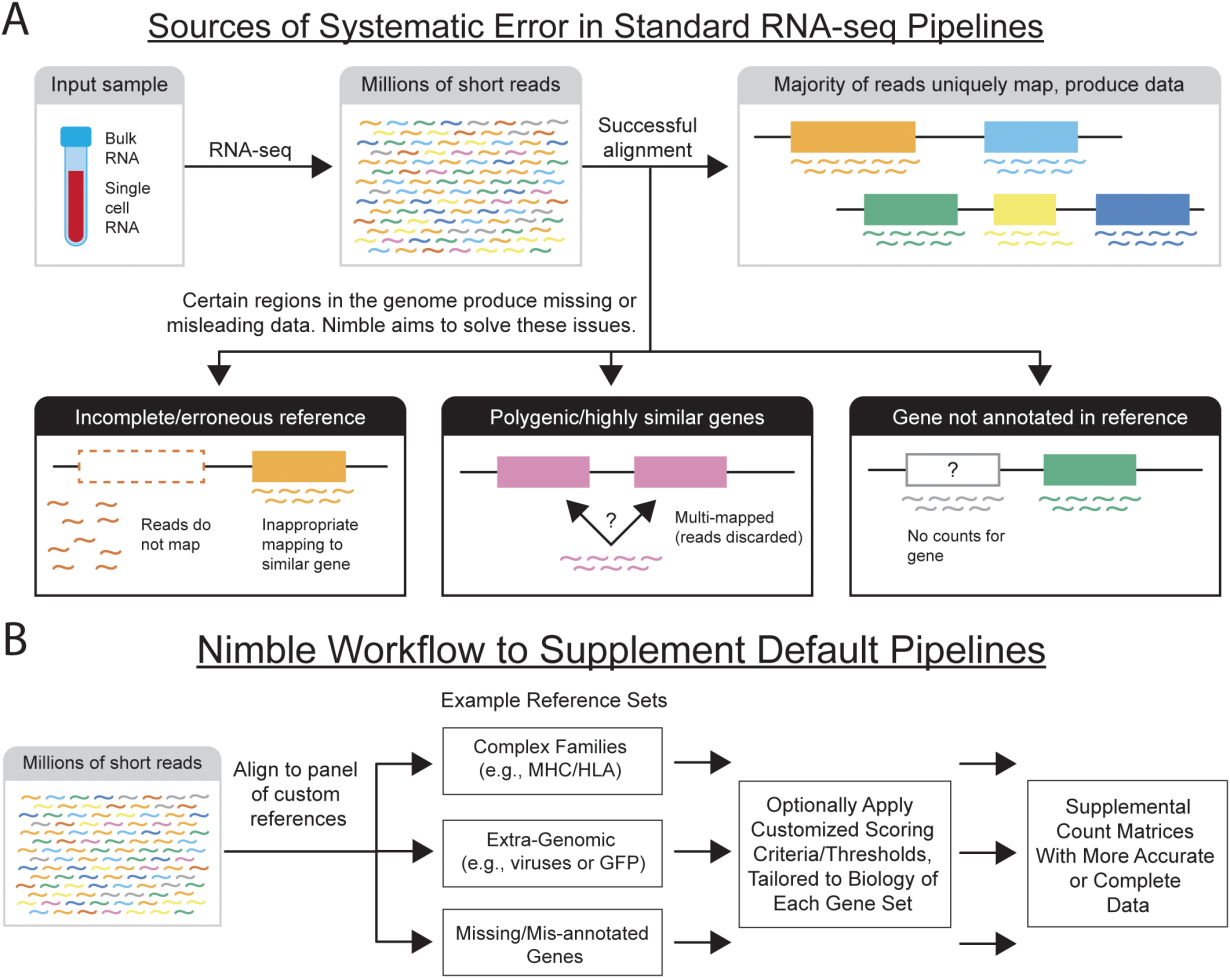
Diagram of RNA-seq alignment and potential pitfalls. **(A)** The schematic illustrates the alignment of short read data to a reference genome. In most cases, the short reads uniquely map to that reference, providing unambiguous gene counts. There are nonetheless several examples, with both technical and biological cases, that result in reads being unable to uniquely map to the genome, resulting in either missing or inaccurate gene counts. If the reference assembly is incomplete and lacks a gene, or if that gene is present but not annotated, either missing data or inaccurately aligned counts are produced. Further, when multiple copies of highly similar genes are present, the aligned often cannot uniquely assign the reads to a gene, often resulting in lost data. Finally, certain regions of the genome are highly polymorphic across the population (e.g., MHC) making it virtually impossible to accurately represent the species using one reference genome. **(B)** The schematic illustrates the workflow used by nimble to address deficiencies in standard single-cell and bulk RNA-seq pipelines. Nimble allows the user to create multiple custom reference spaces, where each is generally designed to address a specific need, such as a reference containing the sequences of genes missing or mis-annotated in the reference, or extra-genomic sequences (e.g., a viral genome). This could also involve specialist databases, such as a reference containing all MHC/HLA alleles. Equally important, nimble allows customized feature calling thresholds for each reference. This is critical to support applications like MHC-typing, where higher resolution matches are required than standard feature calling. The result of this pipeline is a set of supplemental count matrices containing per-sample or per-cell counts for the additional genes/features. These data can either be merged with the existing gene counts, or analyzed in parallel, depending on the experimental needs.

There are nonetheless situations where standard pipelines are systematically inaccurate or sub-optimal (**Figure 1**). Complex regions of the genome, especially gene families with copy number differences and/or segmental duplication, are difficult to accurately assemble when generating reference genomes. If the reference genome is inaccurate or incomplete, this results in feature counting artifacts, such as missing counts for expected genes. If a gene that is transcribed is not represented in the reference genome, the RNA-seq reads from that gene can misalign to the closest available gene, inflating these counts and providing misleading data. Improved genomic assemblies, especially those generated from long read sequencing, will improve this to a point; however, there are gene families with characteristics that remain problematic. Instances where two highly similar genes are encoded in the genome can result in alignment ambiguity or multi-mapped reads, which are often discarded, resulting in lost data. Some gene families have high degrees of variation between individuals, meaning it is extremely difficult to represent genomic diversity of the species with one single reference genome. Examples of these include the major histocompatibility complex (MHC), which is the most variable region of primate genomes, or killer immunoglobulin-like receptors (4–9). In the case of MHC class I, in addition to variable gene content in some species, there is extremely high allelic diversity, with thousands of known alleles (5). While most RNA-seq and scRNA-seq analyses are designed to ignore allelic variation, the identity of the expressed MHC alleles for a subject is critical to antigen recognition, and thus higher resolution genotyping is often needed. Standard RNA-seq pipelines generally treat quantification of all genes identically, which does not permit adaptation of feature calling to match the biology or differing needs of certain gene families.

To address the limitations of standard RNA-seq and scRNA-seq pipelines, we developed nimble, a lightweight tool intended to provide supplemental gene counts to complement standard pipelines (**Figure 1**). Nimble is designed to be executed against one or more customizable gene spaces, where each gene space contains a focused set of reference sequences to address a specific question. Nimble uses a previously published pseudoalignment engine to align reads against these references (10), followed by customizable logic for feature calling. The combination of these two capabilities allows nimble to quantify both simple and complex gene families, especially when the biology or characteristics of these genes are problematic for the standard one-size-fits all alignment and feature calling pipelines.

## Results

### Design of nimble and concordance with standard pipelines

**Figure 2:**
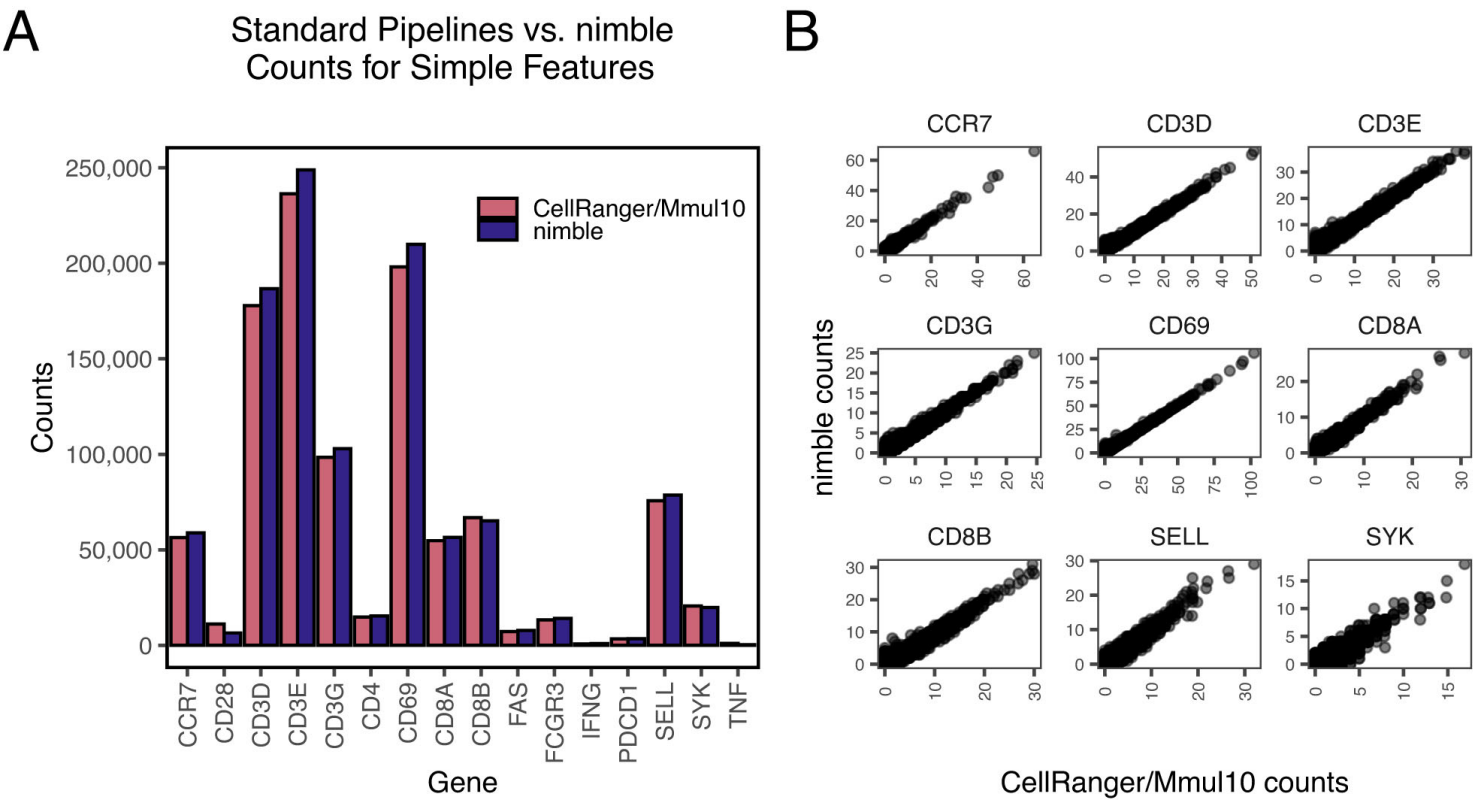
Validation of nimble accuracy relative to established pipelines. All panels display summaries of raw count data generated by processing rhesus macaque scRNA-seq data through either nimble (using a gene space comprised of the mRNA sequences for these genes), or CellRanger (using the Mmul10 reference genome). **(A)** The bar plot compares the magnitude of raw counts generated by nimble and CellRanger/Mmul10 in aggregate for a set of common immune genes. These genes were selected because they represent “simple” features, present at a single copy in the genome without complex polymorphism expected between subjects. **(B)** The scatter plot compares the range of counts generated by nimble and CellRanger/Mmul10 for each cell in the dataset. These data demonstrate that both tools produce similar per-cell counts when executed against typical genes.

Nimble is the combination of a previously developed pseudoaligner and customizable feature-calling algorithm, designed to allow the user to perform targeted quantification of one or more panels of interest. While nimble is primarily designed to address complex genomic regions, we first constructed a panel of “simple” genes that lack the complex genetics or high intra-species variation that can confound standard RNA-seq pipelines. We processed a single-cell RNA-seq (scRNA-seq) dataset from rhesus macaque peripherical blood mononuclear cells (PBMC) and compared the counts obtained by the CellRanger pipeline using the Mmul10 reference genome (“CellRanger/Mmul10”) against the counts obtained by nimble using this custom gene space (**Figure 2**). We contrasted un-normalized raw counts in aggregate, prior to downstream normalization or other processing, to provide the most direct comparison of alignment behavior. The results are highly similar both when comparing the total counts per gene (**Figure 2**), and the per-cell counts (**Figure 2**). While there is minor variation between the tools, this is likely due to differences in alignment algorithm or scoring thresholds. While nimble is not designed to completely replace standard alignment and feature calling pipelines, to provide a more comprehensive comparison of nimble with standard pipelines we generated a nimble library containing the complete 15,782 genes defined in the MMul_10 genome, and compared the resulting per-cell counts against the same data processed with CellRanger/MMul_10. The results were highly concordant, with a Pearson correlation of 0.968 (**Supplementary Figure 1**). Together, these data indicate that nimble’s alignment pipeline captures similar count data to standard pipelines, establishing nimble’s accuracy when aligning to a straightforward gene space. Nimble’s performance scales with available hardware via thread-level parallelism and will attempt to fully-saturate the provided cores. RAM usage is low, requiring memory only for the reference de Bruijn graph and 50 UMIs of buffered data from the input.bam file. In one example, aligning 491 million paired-end reads to a ~2,200-feature MHC reference completed in 225 minutes on 18 CPUs, sustaining ~36,000 reads/sec. Performance scales in a nearly linear manner with CPU count.

### Quantification of genes missing from the reference genome enhances measurement of B cell class switching

**Figure 3:**
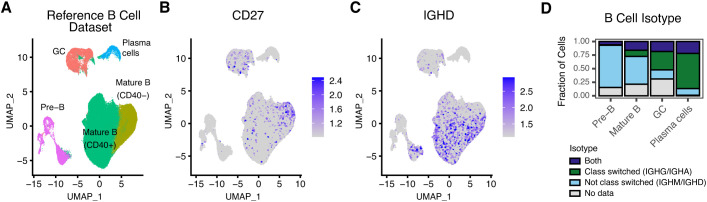
Nimble can quantify features missing from a reference genome. All panels display the analysis of scRNA-seq data from a reference rhesus macaque B cell dataset (19). **(A)** The UMAP displays a dimensional reduction of the reference B cells, colored by B cell subtype. **(B)** The same UMAP as **(A)**, colored by the expression of *CD27*, a gene encoded by rhesus macaques, but not annotated in the Mmul10 genome. **(C)** The same UMAP as **(A)**, colored by the expression of *IGHD*, another feature not annotated Mmul10. **(D)** The *IGHD* count data illustrated in **(C)** was combined with count data from the other Ig heavy chains to categorize cells by isotype. The bar plot displays the result of this classification, grouped by subset. Collectively, these data illustrate situations where nimble can generate missing data to augment standard processing pipelines.

A second straightforward usage of nimble is to quantify genes or features not annotated or misannotated in the reference genome. While this is less common for the human genome, the genomes of model organisms frequently have less complete or accurate gene models. While gene models can be corrected, generating counts for missing features, at least for most scRNA-seq pipelines, requires repeating the entire alignment. Rhesus macaques encode both *CD27* and immunoglobulin heavy constant delta (*IGHD*), and while the sequence for these genes is present in the MMul_10 genome, neither are annotated in the NCBI gene build (version 103). Both genes provide useful information about B cell differentiation states (11). To overcome this, we generated a nimble reference containing these genes, along with the remaining Ig heavy chains (*IGHA, IGHE, IGHM, IGHG1, IGHG2, IGHG3, and IGHG4*) to provide a comparison against standard pipelines (**Supplementary Table S1**). We processed a previously published rhesus macaque reference B cell dataset using this reference space (**Figure 3**). This dataset contains B cells of multiple differentiation states, including Pre-B cells, mature B cells, germinal center (GC), and plasma cells (**Figure 3**). Nimble successfully generated missing count data for *CD27*, demonstrating expression primarily in the “innate-like” *CD40* ^-^ mature B cell cluster, with limited expression among GC cells (**Figure 3**). IGHD is upregulated primarily in pre-B cells and the mature B cell cluster (**Figure 3**). Finally, we used the nimble immunoglobulin heavy chain expression data to classify B cell class switching status (**Figure 3**). For each B cell maturation type, we observe a predominant class-switching status: pre-B and mature B cells were predominately not class switched, while germinal center and plasma cells were predominately class-switched, reflecting their antigen-exposed state. Additionally, as cells transition through the class switch recombination process, we observe many different “mixed” expression states, at a diminished ratio compared to cells that fall into one of the two main class-switching categories. Organizing the cell categories by stage in the B cell maturation and differentiation process, we see the expected transition of B cells from not class-switched to class-switched over time. Taken together, these data demonstrate a case where a new nimble gene space allowed for cell classification beyond what is possible using standard pipelines alone.

### Quantification of extra-genomic features

Many experiments require the quantification of features not encoded by the normal species genome, including the sequences of viral or bacterial pathogens, or exogenous genes (e.g., GFP). A common way to address this situation today is to append the exogenous sequence(s) to the species genome and align data to this new composite “genome”. While this is a viable option much of the time, alterations to the base genome can require re-processing of data, creates issues recombining or merging cohorts (it is more complex to merge counts when not aligned to the identical genomic space). Nimble provides an option to rapidly generate counts for any number of custom features, at any point after the primary alignment is performed, which can either be merged to the primary count matrix or treated separately.

**Figure 4:**
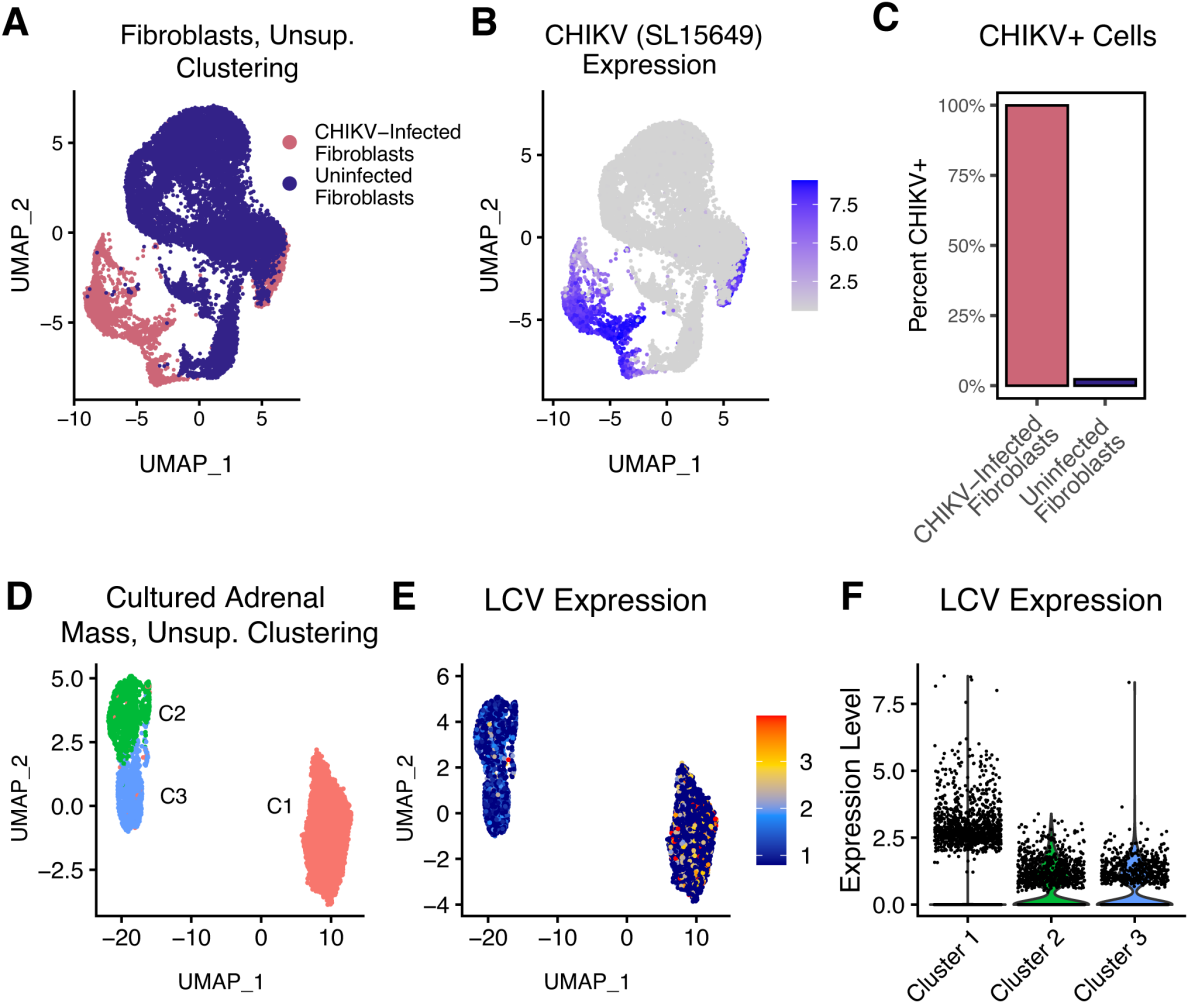
Quantification of viral RNA to illustrate detection of extra-genomic features. Panels **(A–C)** display the results of primary human fibroblasts infected with CHIKV at high MOI, followed by scRNA-seq. Uninfected fibroblasts were included as a control and processed in a physically separate lane. **(A)** The UMAP displays a dimensional reduction of these cells, colored by infection status. **(B)** The same UMAP as **(A)**, colored by nimble-generated quantification of CHIKV RNA, demonstrating that CHIKV is specifically detected in CHIKV-infected fibroblasts and absent in uninfected controls. **(C)** The bar plot quantifies the percentage of CHIKV-positive cells for each fibroblast population from **(A)**. Panels **(D–F)** display scRNA-seq data generated from B cells obtained from an adrenal mass identified in a cynomolgus macaque. **(D)** The UMAP displays a dimensional reduction of adrenal mass-derived B cells. **(E)** The same reduction as **(D)**, colored by nimble-generated LCV expression data. LCV is a ubiquitous opportunistic virus that infects B cells and can induce lymphoma. **(F)** The violin plot quantifies the same nimble-generated LCV expression data shown in **(E)**, demonstrating upregulation of LCV in B cell cluster 1. Collectively, these data provide two examples where nimble provides a simple solution to quantify transcripts not encoded by the host genome.

To demonstrate examples of this, we analyzed virally infected cells. First, we performed scRNA-seq on primary normal human dermal fibroblasts experimentally infected with Chikungunya virus (CHIKV; strain SL-15649), as well as uninfected controls (**Figure 4A**). These were processed on separate lanes, and therefore the CHIKV-exposure status of each cell is known. We began by processing using the standard CellRanger/Mmul10 pipeline. PCA/UMAP analyses revealed two main transcriptional clusters, which largely separate the CHIKV-infected from uninfected cells (**Figure 4**). We aligned these populations to a custom nimble gene space containing the CHIKV genome, generating per-cell counts. CHIKV-expression corresponded extremely well with the expected groups, with virtually all CHIKV-exposed cells expressing high levels of CHIKV and no CHIKV detected in the control cells. This indicates that nimble is accurately and specifically detecting CHIKV (**Figures 4B**).

Because the CHIKV experiment involved experimental viral infection, it was obviously important to quantify CHIKV, and the proper CHIKV reference sequence was known prior to analysis. Therefore, alignment of data to an augmented genome and using standard pipelines would be as effective as nimble. This situation is not always true. Next, we performed scRNA-seq on cells cultured from an adrenal mass detected in an immunosuppressed cynomolgus macaque (12). We hypothesized that a virus was the cause of adrenal mass, but this was not known prior to the experiment. We used nimble to align the reads against a genome containing multiple macaque viruses (**Supplementary Table S1**). Unsupervised clustering on RNA expression (using the host genome and not viral transcripts), revealed multiple transcriptional clusters (**Figure 4**). The nimble-generated data identified lymphocryptovirus (LCV) in B cells isolated from this sample (**Figure 4E**). LCV is a member of the gammaherpesvirus family that naturally infects macaque populations, infects B cells, and can cause lymphomas, especially in immunocompromised subjects (13, 14). Also of note, the per-cell expression of LCV differed between the clusters (**Figure 4**). This is noteworthy because LCV RNA was not part of the dimensional reduction, and therefore the pattern of clustering is driven by changes in the host transcriptome alone. LCV has complex interactions with the host cell, and this suggests that the host gene expression differences could be the effect of increased viral replication or represent different phases of the viral life cycle. While in this experiment we quantified LCV as a single feature, allowing assignment of cells as LCV-positive or LCV-negative, it would be possible to repeat this nimble analysis using a gene space containing the individual LCV transcripts for a more precise quantification. Together, these examples demonstrate that nimble can generate specific quantification for extra-genomic features, such as viruses. It also provides an example where iterative alignment against custom gene spaces could be advantageous over modifying the base genome and repeating the entire analysis.

### Resolution of complex, multigenic families such as NKG2 and KIRs

**Figure 5:**
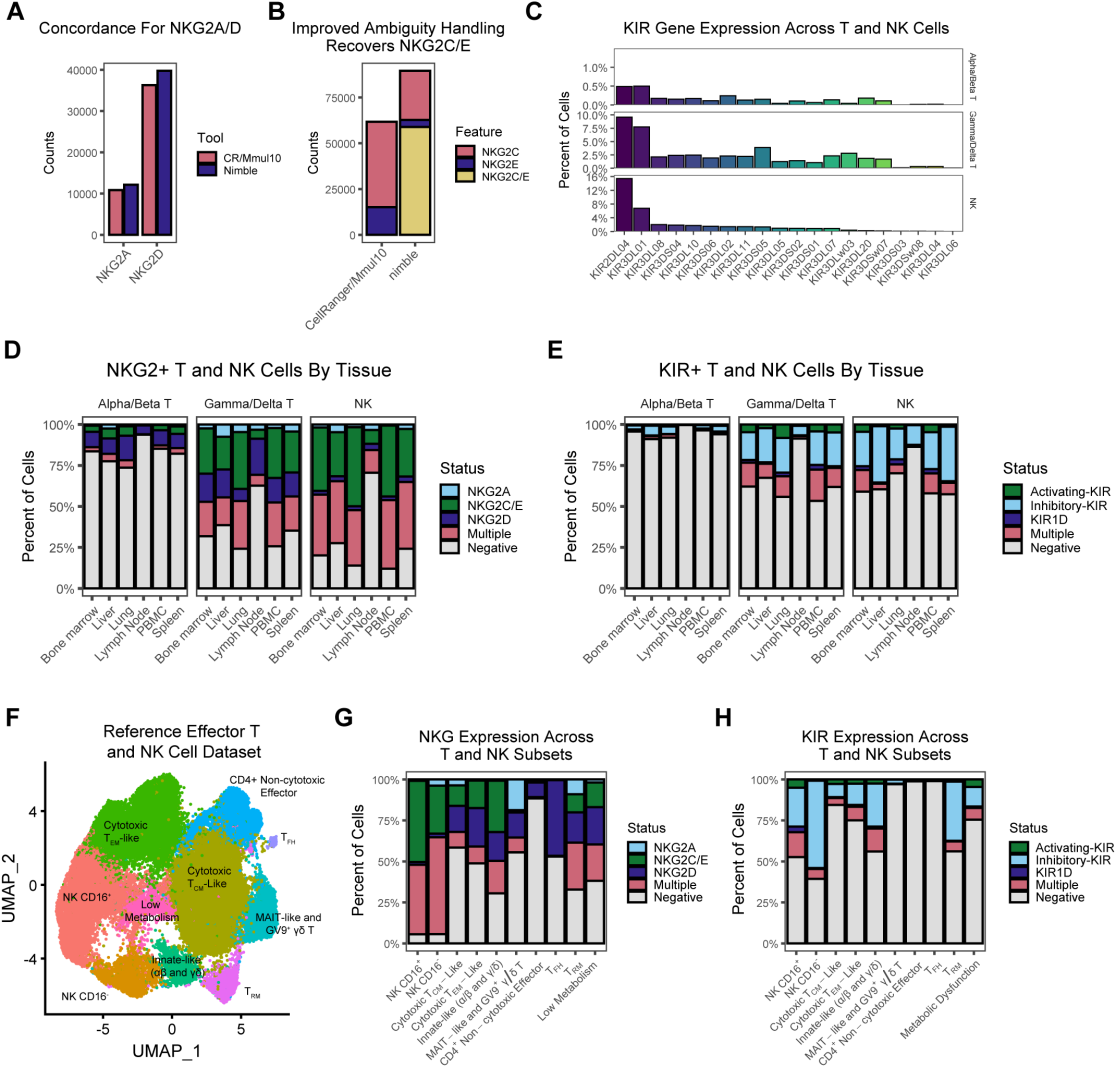
Summarization of NKG and KIR expression across T and NK populations. **(A)** The bar plot displays the magnitude of aggregated counts for *NKG2A* and *NKG2D*, demonstrating that nimble and CellRanger/Mmul10 exhibit similar behavior for these two features. **(B)** The bar plot displays the magnitude of aggregated counts for *NKG2C*, *NKG2E*, as well as reads that mapped to both *NKG2C* and *NKG2E*, which are two functionally similar genes with high sequence similarity. Critically, the difference in ambiguity resolution strategies between CellRanger/Mmul10 and nimble leads to a significant disparity in the number of counts generated for these features. Because nimble can be configured to retain and report ambiguous results, we can recover more counts for the activating NKG2C/E case than standard pipelines. **(C)** The bar plots display the percentage of cells across the RIRA T and NK cell types that are positive for various KIR genes. **(D)** The bar plots display the percentage of RIRA T and NK cells that express nimble-generated NKG data across various RIRA tissue types. **(E)** A similar set of bar plots as **(D)**, displaying the percentage of RIRA T and NK cells that express nimble-generated KIR data across various RIRA tissue types. **(F)** The UMAP displays a dimensional reduction of a population of effector T and NK cells colored by RIRA subtype. **(G)** The bar plot represents the percent of cells that express NKG across the clusters we defined in **(F)**. **(H)** A similar bar plot to **(G)**, representing the percent of cells that express KIR across the clusters we defined in **(F)**.

The examples shown thus far would be possible using standard scRNA-seq analysis pipelines, although there are situations when it might be more convenient or flexible to generate these data using nimble. There are nonetheless many gene families, particularly those with gene duplication or variable copy number, where aligning data and calling features in one-size-fits-all logic creates artifacts. When aligning RNA-seq data to a reference, an important technical decision point, which is often obscured from the end-user, is whether to discard alignments that are mapped to multiple features. These ambiguous “multi-mapped” reads are often discarded in standard pipelines, which can result in the systematic loss of biologically important data. The NKG2 genes are a family of cell surface receptors expressed on NK cells and a subset of T cells (15, 16). This includes the inhibitory receptor NKG2A and the activating receptors NKG2C, and NKG2E. In concert with CD94, these receptors can recognize MHC-E ligands (16, 17). NKG2D is a separate activating receptor that binds numerous MHC-I ligands (17, 18). These receptors are an important part of NK cell signaling and can modulate T cell signaling and thus quantifying these is important for the study of these cells. As is common among many expanded families of genes, there is a high degree of sequence similarity between NKG2C and E, which has the potential to result in multi-mapping of reads and loss of data. To examine the role of multi-mapping in standard RNA-seq pipelines, we obtained a reference scRNA-seq dataset consisting of T and NK cells from eight tissues and 47 rhesus macaques (19). We processed these data using the standard CellRanger/Mmul10 pipeline, along with nimble using a custom gene space containing NKG2 and KIR genes (**Supplementary Table S1**). Further, because we understood the biology of these genes differed from the genome as a whole, we executed nimble in a mode to allow and report multi-feature hits for these gene families. For NKG2A and NKG2D, which are both single-copy and have relatively unique sequences, we observe high concordance between nimble and the standard CellRanger/Mmul10 pipelines; however, there is a significant difference for NKG2C and NKG2E (**Figure 5**). Because NKG2C and NKG2E have high sequence similarity, many short Illumina reads will match both genes and no aligner will be capable of uniquely differentiating them; however, because NKG2C/E are functionally related activating receptors, there can be value in capturing and quantifying those ambiguous hits, especially if this results in a significant increase in data. This is exactly what we observe: by tolerating and reporting multi-mapped data, nimble recovers 45% more counts than the standard pipelines (**Figure 4**).

In additional to the NKG2 family, the killer immunoglobulin-like receptors (KIRs) are a well-characterized polygenic gene family also involved in NK and T cell signaling (7, 20, 21). KIRs are polygenic, with 23 genes present in rhesus macaques, which can be divided into activating and inhibitory KIRs (20, 21). This raises similar alignment/ambiguity issues as NKG2C/E; however, KIRs present a second layer of difficulty for standard pipelines. The gene number and gene content are variable between KIR haplotypes, which is a challenge when trying to use a ‘one-size-fits-all’ genome to represent the entire species. Because the reference genome represents one possible haplotype, individuals will encode different configurations of KIRs than represented in the reference. Further, polygenic regions with segmental duplications are notoriously difficult to sequence and are often poorly represented in reference genomes. The Mmul10 genome contains only 11 KIR genes and thus cannot provide accurate data for this gene family. We processed the same T and NK data using a gene space containing all published rhesus macaque KIR sequences (**Supplementary Table S1**). This reference gene space contained allele-level sequences; however, we executed nimble in a mode to aggregate results to the KIR gene level (**Figure 5**). As expected, KIRs are primarily expressed in NK cells, although expression was detected in gamma/delta (γδ) and to a lesser degree alpha/beta (αβ) T cells. KIR2DL4 and KIR3DS1 were the most detected KIRs. Collectively, these data illustrate the ability to nimble to recover data missed from standard pipelines by employing a more complete reference gene space and executing feature calling using logic more appropriate to the biology of the target genes.

### Characterization of NKG2 and KIR expression in NK and T cells

The enhanced NKG2 and KIR data obtained by nimble allow more detailed characterization of the expression patterns of these functionally important receptors. Because the data in **Figure 5** are derived from a comprehensive single-cell atlas, they provide an ideal dataset in which to characterize expression patterns (19). Expression for NKG2 and KIR genes was variable by tissue, although this likely reflects the composition of the T and NK cells at these sites. For example, the lowest NKG2 and KIR expression was detected in lymph nodes, which are sites dominated by naive and central memory cells (**Figure 5D**). To resolve expression in more detail, we subset to effector-differentiated cells and performed dimensionality reduction (**Figure 5**). The subsets defined in **Figure 5** are explained in greater detail in the original publication (19). As expected, the NKG2 and KIR expression is more common in NK cells relative to T cells. Most NK cells are positive for NKG2A, NKG2C, or both (**Figure 5**). CD16+ NK cells, which are more cytotoxic, have a higher fraction of NKG2C+ cells relative to the CD16- NK subset. T cells were more likely to be NKG2D+ without NKG2A/C/E; however, approximately 10% of effector memory T cells (T_EM_) express NKG2C/E. T follicular helper (T_FH_) cells had the highest fraction of NKG2D+ cells. Gamma/delta T cell subsets showed intermediate NKG2 expression relative to NK cells and αβ T cells, with MAIT-like T αβ cells and TRGV9+ γδ T cells being the population with the largest fraction of NKG2A+ cells. KIR expression was also primarily detected in NK cells (**Figure 5**). Cells are more likely to express inhibitory KIRs alone, and a higher fraction of CD16- NK cells expressed KIRs relative to the CD16+ NK cells, the opposite of NKG2 expression. Among the T cell subsets, γδ T cells had intermediate levels of KIR positivity, and KIR expression was nearly absent from T_FH_ cells, and KIR expression is relatively rare in most αβ T cells. A notable exception was tissue resident memory T cells (T_RM_), which are αβ T cells differentiated for rapid response to antigenic stimulation (22–24). Nearly a third of these cells expressed KIRs, which is a significant difference from other αβ T cell subsets and even higher than CD16+ NK Cells. In total, these data illustrate additional information that can be gleaned from existing scRNA-seq datasets, by combining standard RNA-seq pipelines with targeted alignment and feature calling that are adapted to the biology of complex gene families.

### Quantifying major histocompatibility class I and II allelic expression

**Figure 6:**
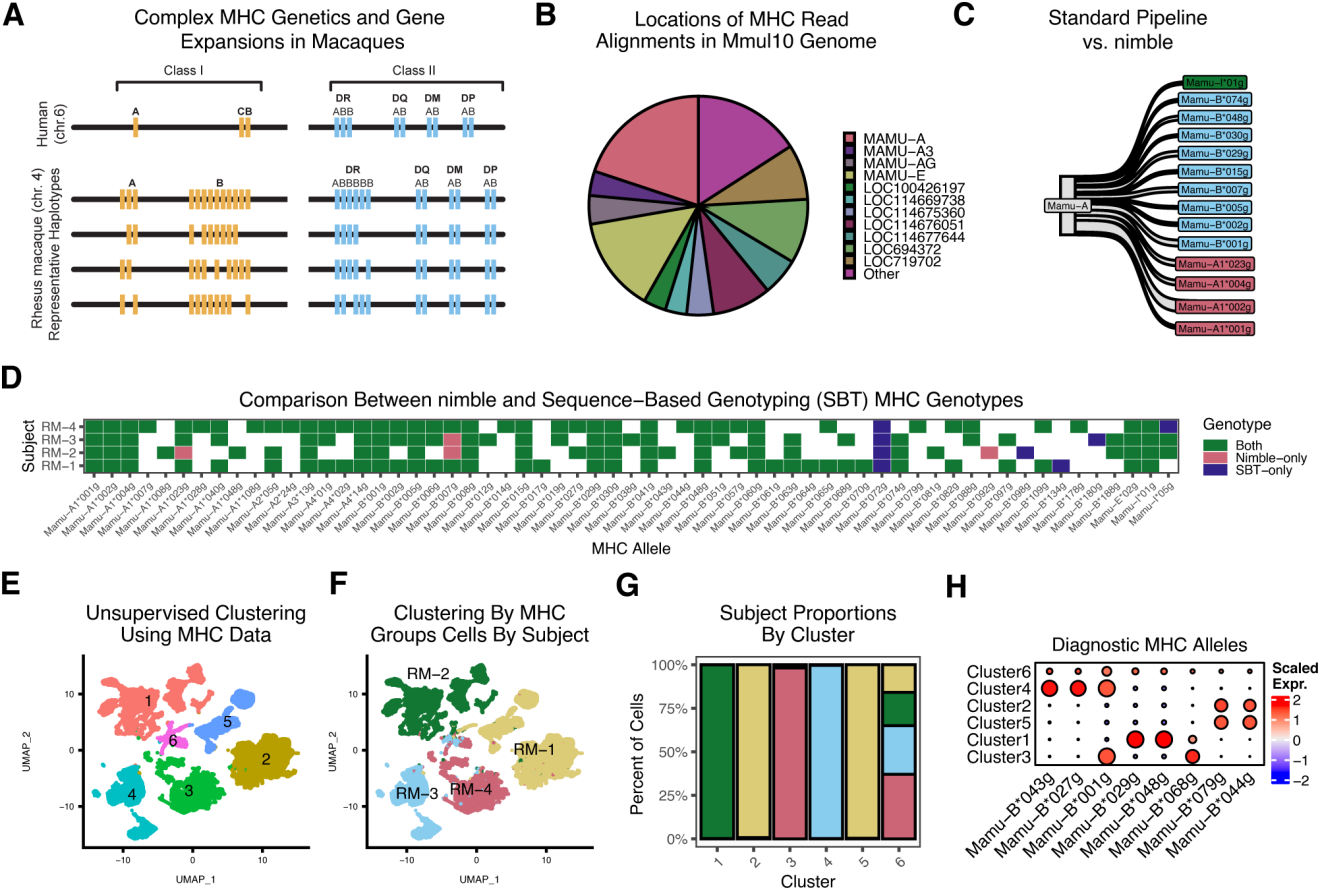
High-resolution MHC genotyping and quantification using nimble. All panels summarize scRNA-seq data obtained from sorted T cells of four rhesus macaques. **(A)** A schematic of the MHC region, illustrating the hypervariability of the region. **(B)** The pie chart summarizes the alignment status for all MHC reads, as assigned by the standard CellRanger/Mmul10 pipeline. Because the Mmul10 genome only contains a handful of MHC-I or MHC-I-like genes, any read with sufficient sequence similarity will map to one of these loci. Note, the genes with “LOC” designations indicate a gene that was not assigned a formal name in the NCBI gene build. This is both a reflection of the incomplete MHC sequence present in the Mmul10 genome, and the imprecision of count data with respect to the MHC. **(C)** The Sankey plot summarizes all reads assigned to Mamu-A by the CellRanger/Mmul10 pipeline, which is compared against the higher resolution MHC genotype data generated by nimble. While many reads are from Mamu -A, where nimble simply reports a higher resolution genotype, a significant number of the reads assigned to Mamu-A are from Mamu-B alleles, highlighting the inaccuracy of MHC data from standard pipelines. **(D)** The tile plot summarizes the concordance between nimble-generated data and MHC typing data generated on the same animals using an independent sequence-based genotyping (SBT) assay. Panels E-H provide a proof-of-concept example to illustrate how MHC typing data can be used to demultiplex pooled cells from scRNA-seq experiments. **(E)** The UMAP displays a dimensional reduction computed from nimble-generated MHC allele data across the same subjects as shown in **(C)** and **(A)**, colored by unsupervised cluster identity. **(F)** The same UMAP as **(E)**, colored by known subject identity. **(G)** The bar plot shows the proportion of cells within each unsupervised cluster assigned to each subject. Together, **(E–G)** indicates that MHC allele expression per-subject is relatively unique, and that using it to perform unsupervised clustering recovers many subject-specific clusters. **(H)** For the heatmap, we compute a gene module per-subject by getting the top differential genes by cluster, indicating that each subject has a set of unique MHC alleles that uniquely identify them, thereby driving cluster differentiation.

The major histocompatibility complex (MHC) is among the most polymorphic in the genome and presents multiple challenges for traditional RNA-seq analyses. Because of the high importance of MHC/HLA genotyping and the unique challenges, an entire field has emerged dedicated to MHC genotyping (5). The MHC is divided into class I and II loci. In humans, there are three MHC class I loci (termed human leukocyte complex or HLA): HLA-A, -B, and –C; however, in macaques there are a variable number of MHC-A and –B loci, ranging from 20–24 distinct alleles per macaque (**Figure 6**) (25). The latter creates similar challenges as KIRs and presents a major problem for reference genome design. Further, most MHC loci have extremely high allelic diversity, with thousands of unique alleles per species (5). These allele-specific polymorphisms alter the peptide binding potential of MHC alleles, meaning that genotyping at high resolution is required. This is quite distinct from the approach used for RNA-seq quantification of most genes, where pipelines are generally designed to ignore subject-specific polymorphism within a gene. Together, these present twin problems: 1) the single reference genome cannot adequately represent species-level diversity, and 2) genotyping at a much higher resolution is required than for typical genes.

To demonstrate the ability of nimble to generate high-resolution and accurate MHC typing from scRNA-seq data, we processed scRNA-seq data from four rhesus macaques against a reference space with 2,379 MHC-I and MHC-II alleles (**Supplementary Table S1**). Unlike prior figures, nimble was run in a mode to report only perfect sequence matches, which is essential for the MHC, where nucleotide differences as little a single base pair change alter peptide binding potential, necessitating high-resolution allele-level genotyping. While the database contained all known rhesus macaque MHC alleles, the resulting data were summarized by lineage (e.g. two-digit typing) (5, 26). We began by isolating the raw sequence reads nimble detected as aligned to MHC and compared the nimble result against the CellRanger/Mmul10 alignments. As noted above, the Mmul10 genome is incomplete and inaccurate across the MHC region, due to the high complexity and polymorphism of that region. It nonetheless contains a handful of genes annotated as MHC or MHC-like. The majority of reads nimble identified as MHC were aligned to these loci (**Figure 6**). While MHC loci are highly polymorphic, polymorphism is clustered in the peptide-binding region with most regions of the gene being much more conserved. Because the Mmul10 reference genome only contains a handful of MHC loci, and these loci do not represent the diversity of the species, all reads from MHC will pile up against the best available target. This is shown in greater detail when comparing the reads assigned to the gene annotated as MHC-A in Mmul10 against the higher resolution genotyping generated by nimble (**Figure 6**). This shows that while many of the reads assigned to MHC-A by standard pipelines are MHC-A alleles, a significant number of MHC-B alleles are also aligned to this gene. This highlights the fact that standard pipelines generate incorrect data from complex loci, in large part because they are simply not designed to differentiate this level of complexity. To evaluate the accuracy of nimble MHC data, we genotyped the subjects using an alternative sequence-based genotyping (SBT) assay, commonly employed for MHC genotyping of rhesus macaques (27). This assay involves PCR amplification of a small amplicon spanning the most variation portion of MHC-I alleles, using primers conserved across most MHC-I alleles. The genotypes obtained from each assay were overwhelmingly concordant, supporting nimble’s accuracy. The small number of discrepancies can be explained by methodological differences. The most notable was the lack of Mamu-B*072 detection in the nimble/scRNA-seq data. When we inspected the raw nimble results, Mamu-B*072 alignments were detected; however, they were always ambiguous with different MHC alleles and thus discarded by nimble’s featuring calling logic. Because scRNA-seq data randomly samples regions of mRNA molecules, as opposed to the targeted sequencing of SBT, and because many MHC alleles are highly similar, it may not always be possible to resolve every allele. Depending on the goal of the analysis, nimble’s alignment and scoring parameters could be adjusted. These data nonetheless demonstrate a resolution of MHC genotyping far superior to standard pipelines.

### High-resolution MHC typing from scRNA-seq can assign scRNA-seq transcriptomes to subject

Due to cost, it is common to pool samples in single-cell RNA-seq experiments. Multiple methods exist to demultiplex samples, including cell hashing reagents and genotype-based approaches (28–31). Due to the high subject-to-subject diversity of MHC, these MHC genotypes should provide a mechanism to assign cells to subjects as well. To test this, we performed dimensionality reduction and unsupervised clustering on the MHC genotypes generated by nimble, using the same four rhesus macaques, which identified six clusters (**Figure 6**). This method was effective at separating cells by subject (**Figure 6F**). Pairwise differential gene expression analyses between these clusters identified a handful of MHC alleles with cluster-specific expression patterns, which correspond to alleles uniquely expressed by one or two subjects (**Figure 6**). These data illustrate one practical usage for high-resolution MHC genotyping from scRNA-seq data.

### Differential regulation of individual MHC alleles following Mtb lysate exposure

**Figure 7:**
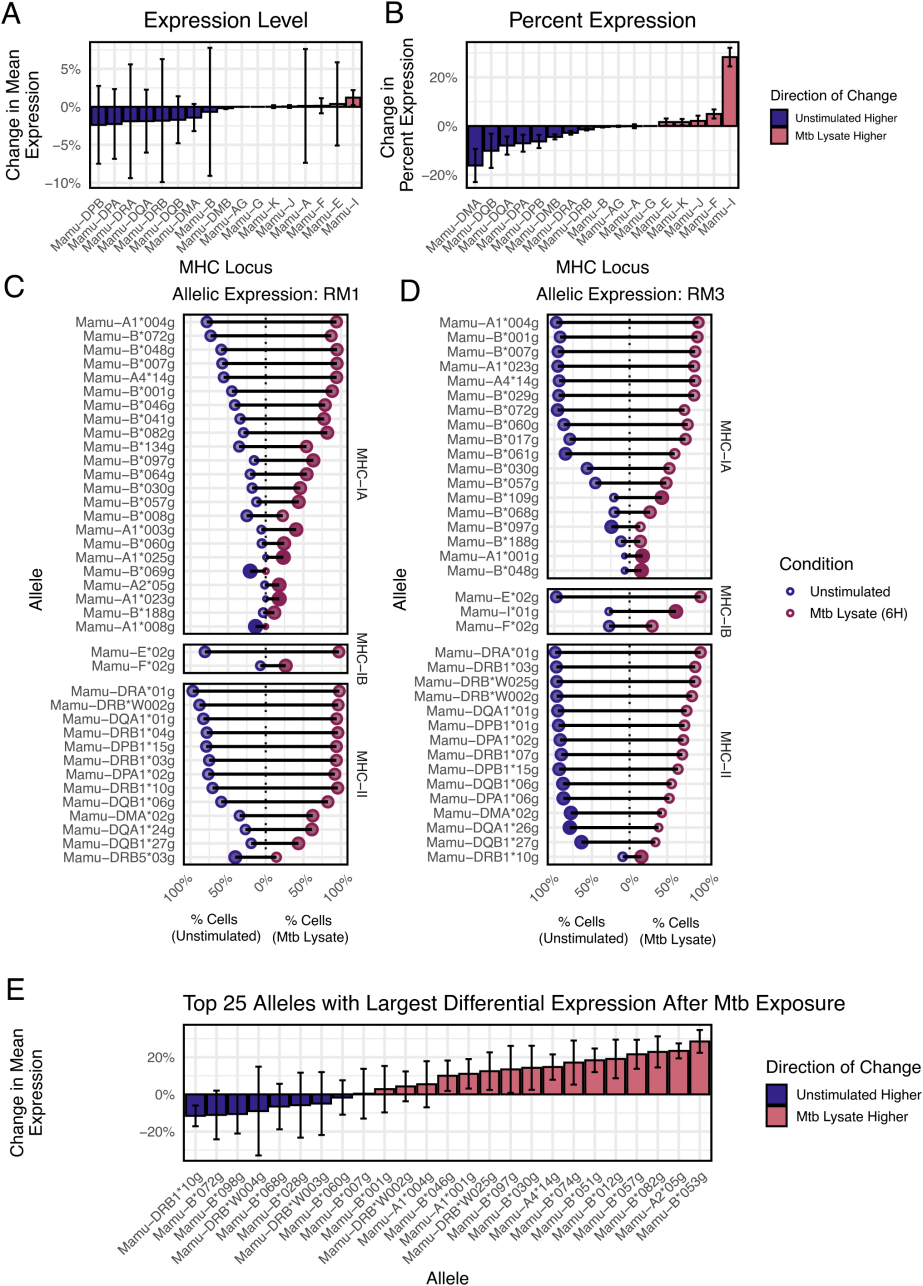
Detection of variance in MHC allele expression between stim and control data. **(A)** The bar plot displays the magnitude and variation of the aggregated expression difference between stim and control data for each MHC locus. **(B)** A similar bar plot to **(A)**, displaying the percentage difference and variance of the number of cells that express each MHC locus, compared between stim and control data. **(C, D)** The dot plots show the top variable alleles for each subject. For each allele, the data are split between stim and control expression. Negative percentage values indicate expression in unstimulated cells, while positive percentage values indicate expression in stimulated cells. The x-axis is the magnitude of the expression for each allele. The dot size and color represent the “skewedness” of the expression toward stim or control data, indicating an upregulation. **(E)** In the bar plot, we rank all MHC alleles across the subjects by mean “skewedness” and display the top twenty-five, allowing us to identify alleles that are systematically upregulated in either the stim or the control data.

MHC expression can be altered in response to pathogens, although most data measure global regulation of all alleles from a given MHC locus, since allele resolution of expression has been difficult to measure (32, 33). To examine this, we generated a dataset with bronchial alveolar lavage cells from a cohort of 12 rhesus macaques vaccinated against *Mycobacterium tuberculosis* (*Mtb)*. The recovered cells were overwhelmingly macrophages. We divided these cells and exposed one fraction to *Mtb* lysate for 6 hours, with the remaining cells serving as a control, followed by scRNA-seq. We used nimble to quantify MHC-I and MHC-II expression levels. First, we quantified MHC at the locus level to identify any systematic changes across a whole MHC locus (**Figures 7A, B**). Data were quantified as either the mean expression level or the percent of cells expressing each locus. Across all subjects, we see changes in mean expression of <5% in *Mtb* stim or control unstimulated cells, with generally high variance, especially for MHC-B, suggesting expression may vary by allele. The percentage of cells expressing MHC loci varies by <10% in *Mtb* exposed or control unstimulated cells, except for Mamu-I. There was a small overall increase in the percentage of cells expressing any MHC-A and MHC-B alleles.

When the data are summarized at the allele-level, a more complex picture emerges (**Figures 7C, D**). First, the per-cell expression of each MHC allele varies heavily both at rest and post-stimulation. Certain MHC alleles are expressed by nearly all cells (e.g., Mamu-A1*004), while some are only expressed by a small fraction of cells (e.g., Mamu-A1*008 or Mamu-DRB1*10). Second, individual alleles behave differently after *Mtb* exposure, with some alleles increasing significantly relative to controls, some unchanged, and some even decreasing (e.g., Mamu−DRB5*03). When summarized across all rhesus macaques, Mamu-B*053 showed the highest increase after exposure, while Mamu-DRB1*10 showed the highest decrease (**Figure 7E**). These data, while proof-of-concept, demonstrate that there is high variability in expression at the level of MHC allele, and that allele-specific regulation occurs. This level of information is largely undetected by standard analysis pipelines. These changes could have implications for antigen selection in vaccines and could contribute to the protective effects of certain MHC alleles.

## Discussion

Dominant RNA-seq and scRNA-seq pipelines are designed to produce reliable quantification across the genome as a whole. Reads are typically aligned to a single reference genome that is intended to represent the genomic diversity of the entire species. While these can be very effective, there are gene families and genomic regions with characteristics that are problematic for standard pipelines, especially polygenic regions with differences in gene content between subjects. These include regions where it is extremely difficult for one reference genome to faithfully represent the diversity of the species (e.g. MHC/HLA or KIR), and situations where the species simply encodes multiple copies of highly similar genes (e.g. NKG2C/E). There are also more mundane situations where standard pipelines can fail or be sub-optimal, including detection of exogenous RNA (e.g., a virus) or simple errors in the gene model. Here, we presented nimble, a novel and flexible tool to address these gaps, especially where the underlying biology doesn’t lend itself to a one-size-fits-all algorithm. Nimble supplements standard pipelines by aligning data to custom gene spaces, with customizable criteria for feature calling, which can be adapted to the needs of that gene family.

We demonstrate the value of nimble through multiple examples. To validate accuracy, we demonstrate concordance between nimble and the standard CellRanger pipeline, using a set of typical single-copy, relatively conserved genes. Nimble can be used to address technical issues in the genome or gene model, as shown for *CD27* and *IGHD*, or for the quantification of viral expression. Nimble is especially powerful for complex, polygenic gene families, because it can perform feature calling using settings more appropriate for each gene family. We show that nimble can recover otherwise discarded expression data for *NKG2* genes and used nimble to characterize *NKG2* and *KIR* expression in T and NK cells. These analyses identified previously unreported enrichment of inhibitory KIR expression in tissue resident memory T cells. Finally, nimble can resolve high-resolution MHC expression data, which revealed significant expression differences at the allele-level, and allele-specific changes after *Mtb* exposure. Collectively, these demonstrate a range of situations where nimble can augment standard RNA-seq pipelines to recover potentially valuable data.

There are many efforts to improve the quality of reference genomes, including the new generation of so-called telomere-to-telomere (T2T) genomes (34, 35). While these may help with some of the issues outlined here, they will not address them all. While newer builds may provide contiguous sequence across the entire chromosome, they still represent the entire genomic diversity of the species with one reference. This is not adequate for highly polymorphic regions, especially when gene content differs between subjects. Newer genomic representations, such as pangenome graphs, seek to represent multiple haplotypes in a single reference and could address some of this (36). While the latter is unquestionably a more biologically appropriate way to represent intra-species variation, this is a significant change, and new generations of software will be required to take advantage of this new genomic representation. Finally, even if a perfect representation of genomic diversity existed, some gene families have characteristics that require customized logic in the scoring of gene and allele-level counts.

Nimble, as the name suggests, is designed to be lightweight and flexible. We presented a set of examples where it is useful, but others may exist. One potential use-case is quantification of isoforms, in which case the reference might contain a handful of isoforms for the gene of interest. This supplemental alignment and count data makes it feasible to identify previously missed patterns of expression across diverse species and cell types.

## Methods

### Nimble aligner

The data presented here were processed using a novel toolchain developed for the purpose of aligning RNA-seq data to arbitrary reference spaces. Nimble provides various facilities for curating these custom reference libraries, aligning sequence data, and reporting properties of the alignment data for the purpose of quality control. The tool takes RNA-seq data in a variety of formats and a set of custom reference libraries as input and produces one count matrix per library. To create a custom gene space, the user can provide a set of Entrez identifiers, a CSV, or a FASTA file. The nimble library file produced allows the user to customize values for aligner filter behavior, such as minimum read length for a passing alignment, the maximum allowable mismatches, or sequence trimming strictness, among several others. The tool and detailed documentation about its usage and configuration options is available on GitHub (https://github.com/BimberLab/nimble).

Nimble incorporates a previously developed, multithreaded pseudoalignment algorithm to align RNA-seq data to these custom gene spaces (10). First, if the input is a.bam file, it is sorted using samtools (37). Then, the custom gene space reference sequences are used to construct a Debruijn graph, which allows for rapid and accurate quantification of counts per input gene without needing to perform expensive positional alignment. All input RNA-seq data is trimmed with a reimplementation of Trimmomatic’s MAXINFO algorithm (38) and aligned to the input gene spaces, producing either counts-per-gene matrices or counts-per-molecule matrices, depending on the format of the input data. Ambiguous alignments to two or more features may be kept or discarded, depending on the user settings.

The nimble alignment pipeline provides several additional layers of filtration for the alignment count matrix, depending on the format of the input data. All alignments are subject to alignment length, mismatch, and trimming filters. In the case of paired-end input reads, there are optional filters for asserting read-pair alignment orientations relative to the reference space, and several options for producing a single set of calls from differing alignments between two sequences in the same read-pair. Finally, nimble can transform the counts-per-molecule matrix produced from 10x scRNA-seq input data into a counts-per-cell matrix by intersecting on the molecule and cell barcodes to conform to the expected data format for downstream packages like Seurat.

### Animal subjects

All study macaques were housed at the Oregon National Primate Research Center (ONPRC) in animal biosafety level 2 rooms with autonomously controlled temperature, humidity, and lighting. Macaques were fed commercially prepared primate chow twice daily and received supplemental fresh fruit or vegetables daily. Fresh, potable water was provided via automatic water systems. During all protocol time points, body weight and complete blood counts were collected and animals underwent anesthesia support and monitoring. The ONPRC Institutional Animal Care and Use Committee approved macaque care and all experimental protocols and procedures. The ONPRC is a Category I facility. The American Association for Accreditation of Laboratory Animal Care fully accredits the Laboratory Animal Care and Use Program at the ONPRC. It has an approved assurance (no. A3304-01) for the care and use of animals on file with the National Institutes of Health Office for Protection from Research Risks. The Institutional Animal Care and Use Committee adheres to national guidelines established in the Animal Welfare Act (7 U.S. Code, sections 2131–2159) and the Guide for the Care and Use of Laboratory Animals, Eighth Edition, as mandated by the U.S. Public Health Service Policy.

### Tissue collection and processing

Cell isolation from PBMC and solid tissues were acquired and processed to single-cell suspensions using previously published methods, summarized below (5, 19). Liver, spleen, and mesenteric lymph node biopsies were collected by a minimally invasive laparoscopic procedure (39). For lung samples, animals were humanely euthanized, and caudal lung lobe samples were collected during necropsy. Bone marrow cells were harvested from the humerus or iliac crest by flushing with R10 media. Peripheral blood mononuclear cells (PBMCs) were isolated from freshly collected ACD-A treated blood utilizing Ficoll-Paque density centrifugation (20). Lymph nodes (LN) and spleen samples were homogenized as previously described (40) while liver and lung samples were enzymatically digested with DNAse and collagenase (41, 42). Prior to processing, cells were filtered using 70 um strainers. Cells were quantified using a Countess II (Thermo Fisher), aliquoted, diluted as required for single-cell RNA sequencing (typically 500-1,500 cells/uL), and kept on ice prior to processing. Mononuclear cells were isolated from bronchoalveolar lavage (BAL) as previously described (43).

### Cell hashing

Cell hashing was used for most scRNA-seq samples, with the MULTI-Seq lipid labeling system (31), using commercially available lipid modified oligos (Sigma Aldridge LMO001). Cells were labeled with barcoded lipids as follows: 15uL MultiSeq solution 1 (LMO stock, diluted in PBS to 400nM) was added, along with 45 uL of the barcode solution (10uM barcode oligo, diluted in PBS to 400nM), giving a final working concentration of 200nM for LMO and 200nM for the barcode oligo. Next, pipet mix and incubate for 5 min at 4°C. Add 10uL of the MultiSeq co-anchor solution (50uM Co-A stock, diluted in PBS to 2uM), then pipet mix and incubate for 5 min at 4°C. Wash twice with 1 mL cold PBS, spinning cells at 700 g for 5 min at 4°C, and then resuspend in 200 uL R10 (which will quench LMOs). Samples were pooled, followed by GEM generation on the 10x instrument.

### Single-cell RNA sequencing

The isolated single cell suspensions were then processed for single-cell RNA sequencing using the 10x Genomics Chromium platform, using 5’ v2 or HT chemistry, following the manufacturer’s protocols, including feature barcoding library preparation. To improve capture of MULTI-Seq fragments, we added the following primer, 5’-CCTTGGCACCCGAGAATTCC-3’, at 2.5uM to the 10x cDNA synthesis step. Generation of VDJ enriched libraries followed manufacturer’s instructions with the exception that macaque-specific TCR constant region primers were used in place of human-specific TCR enrichment primers for macaque cells (19). Primer pairs were used to amplify the alpha, beta, delta, and gamma TCR chains. The concentration of the alpha constant region primer was increased relative to the beta primer to improve amplification. PCR conditions for both reactions were as follows: lid temp 105°C, 98°C 0:45, 12 cycles of: 98°C 0:20, 60°C 0:30, 72°C 1:00, followed by 72°C 1:00 and 4°C hold. Sequence libraries were sequenced using Illumina chemistry, on either Novaseq or HiSeq instruments (Illumina).

### Single-cell RNA-seq processing

Raw sequence reads were processed using 10X Genomics Cell Ranger software (version 8.0.1). The resulting sequence data were aligned to the MMul_10 genome (assembly ID: GCF_003339765.1) with NCBI gene build 103. Cell demultiplexing used a combination of algorithms, including GMM-demux, demuxEM and BFF, implemented using the cellhashR package (30, 44, 45). Droplets identified as doublets (i.e. the collision of distinct sample barcodes) were removed from downstream analyses. We additionally performed doublet detection using DoubletFinder, and removed doublets from downstream analysis (46). Data were otherwise processed as previous described (19). Analyses utilized the Seurat R package, version 4.2 (47). Multiple scRNA-seq datasets are used, including many previously published datasets. A complete listing of the SRA accession numbers for datasets generated for this manuscript are available in **Supplementary Table S2**. Adrenal mass B cell samples were obtained from a previously described case (12). The Rhesus Macaque Immune Atlas (RIRA) dataset was used for multiple analyses, with the input gene expression data from NIH GEO database, accession GSE277821, and NIH BioProject PRJNA1163395 (19).

### Major histocompatibility complex analysis

Genotyping for Major Histocompatibility Complex class I (MHC-I) allele was performed using a PCR amplicon-based method, as previously described ^78,79^. For nimble-generated MHC data, normalization was performed per cell by dividing the raw reads for each MHC allele by the sum of reads from each MHC locus (i.e. total MHC-A, total MHC-B, etc.).

### Chikungunya virus infection

Primary normal human dermal fibroblasts (NHDFs) were experimentally infected with Chikungunya virus strain SL-15649, obtained from Dr. Mark Heise (University of North Carolina at Chapel Hill). NHDFs were plated into 6-well plates, cultured in DMEM containing 10% FBS and 1X PSG, and incubated overnight at 37°C with 5% CO2. Cells were infected in triplicate wells with CHIKV SL-15649 at a multiplicity of infection equal to 1. At 24 hours post infection the cells were trypsinized and washed twice with DMEM-10 and once with PBS.

### Mycobacterium lysate exposure assay

Mononuclear cells isolated from bronchoalveolar lavage (BAL) fluid were incubated at 37°C under a humidified 5% CO_2_ atmosphere. These cells were rested for 4 hours, and then either exposed to Mtb lysate (BEI NR-14822 at 6uL/Test) for 4 hours or cultured without Mtb lysate as a control. After incubation, cells were processed using the 10x Genomic Chromium system, as described above.

## Data Availability

The datasets presented in this study can be found in online repositories. The names of the repository/repositories and accession number(s) can be found in the article/**Supplementary Material**.

## Supplementary material

Supplementary Figure 1 Comparison of nimble to CellRanger for genome-wide alignment. While nimble is not designed to completely replace standard alignment and feature calling pipelines, to provide a more comprehensive comparison of nimble with standard pipelines we generated a nimble library containing the complete 15,782 genes defined in the MMul_10 genome, and compared the resulting per-cell counts against the same data processed with CellRanger/MMul_10. The scatter plot presents the counts for each gene obtained using nimble relative to the CellRanger pipeine. Results were highly concordant, with a Pearson correlation of 0.968 (**Supplementary Figure 1**). Together, these data indicate that nimble’s alignment pipeline captures similar count data to standard pipelines, establishing nimble’s accuracy when aligning to a straightforward gene space.
