# Supplementary material for: An immune-focused supplemental alignment pipeline captures information missed from dominant single-cell RNA-seq analyses, including allele-specific MHC-I regulation
Source: Front Immunol. 2025 Aug 8;16:1596760. doi: 10.3389/fimmu.2025.1596760 (PMC12370678; doi:10.3389/fimmu.2025.1596760)

Supplemental Figure 1. Comparison of nimble to CellRanger for genome-wide alignment. While nimble is not designed to completely replace standard alignment and feature calling pipelines, to provide a more comprehensive comparison of nimble with standard pipelines we generated a nimble library containing the complete 15,782 genes defined in the MMul_10 genome, and compared the resulting per-cell counts against the same data processed with CellRanger/MMul_10. The scatter plot presents the counts for each gene obtained using nimble relative to the CellRanger pipeine. Results were highly concordant, with a Pearson correlation of 0.968 (Supplemental Figure 1). Together, these data indicate that nimble's alignment pipeline captures similar count data to standard pipelines, establishing nimble's accuracy when aligning to a straightforward gene space.

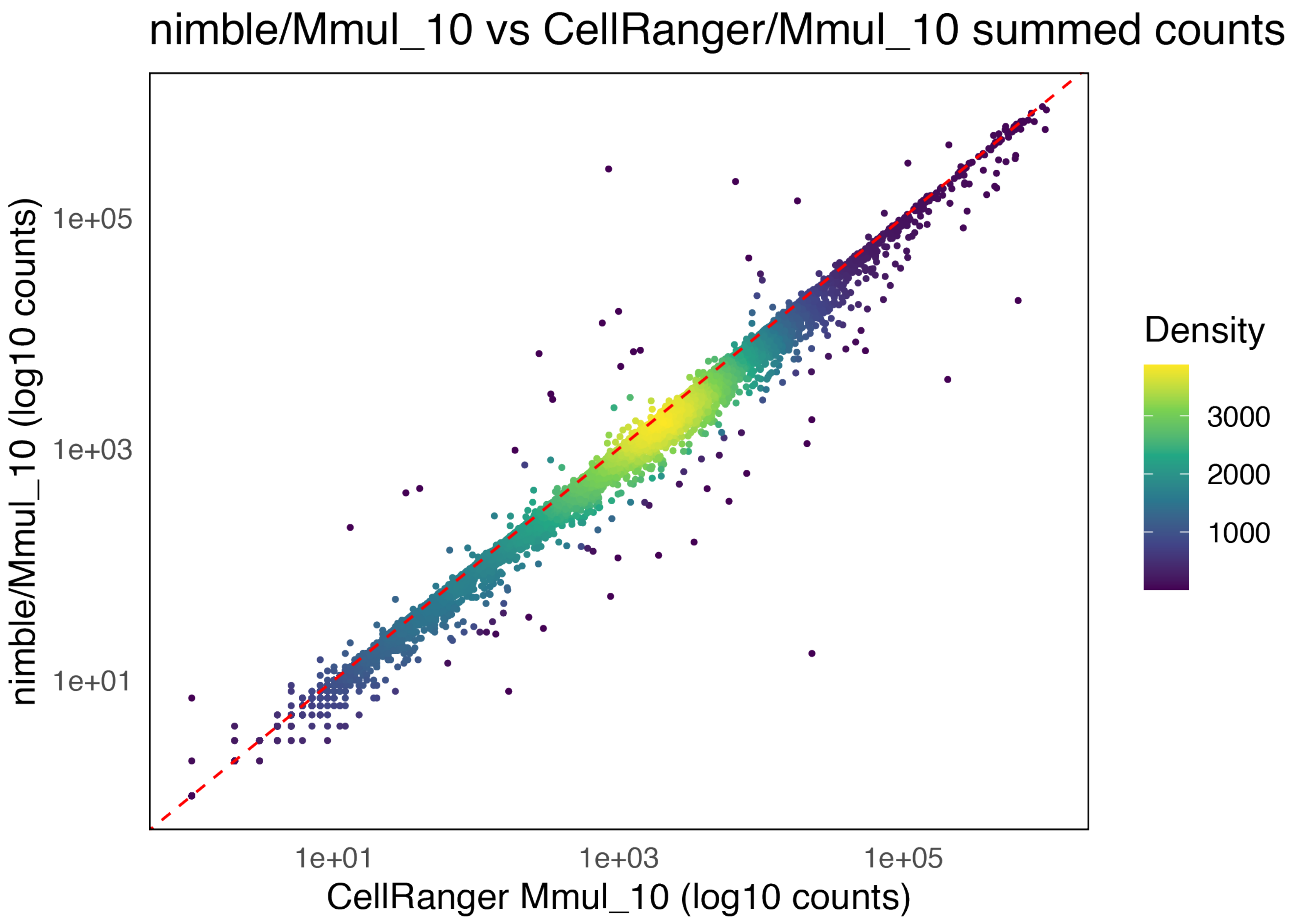

Supplement: Supplementary Figure 1 — Comparison of nimble to CellRanger for genome-wide alignment. While nimble is not designed to completely replace standard alignment and feature calling pipelines, to provide a more comprehensive comparison of nimble with standard pipelines we generated a nimble library containing the complete 15,782 genes defined in the MMul_10 genome, and compared the resulting per-cell counts against the same data processed with CellRanger/MMul_10. The scatter plot presents the counts for each gene obtained using nimble relative to the CellRanger pipeine. Results were highly concordant, with a Pearson correlation of 0.968 ( Supplementary Figure 1 ). Together, these data indicate that nimble’s alignment pipeline captures similar count data to standard pipelines, establishing nimble’s accuracy when aligning to a straightforward gene space. [file Image1.pdf]
